# Lung allograft injury improves after gastroesophageal reflux surgery by donor-derived cell-free DNA kinetics

**DOI:** 10.3389/fmed.2026.1792506

**Published:** 2026-04-01

**Authors:** Samir Sultan, Jon C. Gould, Andrew S. Kastenmeier, Gokulakrishnan Balasubramanian, Niraja Suresh, David J. Ross

**Affiliations:** 1 Medical College of Wisconsin, Milwaukee, WI, United States; 2 Natera Inc., Austin, TX, United States

**Keywords:** aspiration, biomarker, cell-free DNA, chronic lung allograft dysfunction, gastroesophageal reflux, GERD, lung transplantation

## Abstract

Gastroesophageal reflux disease (GERD) and bile acid micro-aspiration are risk factors for the development of chronic lung allograft dysfunction (CLAD) after lung transplantation (LT). Donor-derived cell-free DNA (dd-cfDNA) is a clinically validated plasma biomarker for acute rejection but also detects other types of allograft injury and can potentially be used to detect GERD and micro-aspiration. Here, we report a five-case single-center series of LT patients with confirmed GERD and micro-aspiration, treated by Toupet fundoplication and adjunctive laparoscopic procedures. dd-cfDNA fraction measured pre*-* and post*-* intervention demonstrated a decreasing trend in the state of molecular injury: 3.24% (IQR, 1.675–8.71) vs. 0.75% (0.165–1.825; *p* = 0.0625), respectively. All patients experienced clinical improvement (assessed by spirometry or resolution of recurrent aspiration pneumonia events). These data suggest that dd-cfDNA may have expanded utility as a non-invasive clinical tool to identify molecular injury sequelae of gastroesophageal reflux. Given the limited effective treatment options for CLAD, we envision an opportunity for the use of dd-cfDNA to reduce the risk of CLAD development when GERD is involved.

## Introduction

The long term outcomes after lung transplantation (LT) are suboptimal, with survival rates of 88.5% at 1-year, 71.3% at 3-years, 59.7% at 5-years, and 31.8% at 10-years (1). Chronic lung allograft dysfunction (CLAD) is a highly prevalent form of chronic rejection and the leading cause of mortality after the initial year in both the United States and Europe (2, 3). Gastroesophageal reflux disease (GERD) and bile acid (BA) micro-aspiration are risk factors for CLAD (4). BAs, specifically taurocholic and glycocholic acid, are detectable in bronchoalveolar lavage fluid (BALF) in association with GERD. Further, BA can engender an inflammatory microenvironment, which can then precipitate acute lung allograft dysfunction (ALAD) (5). The diagnosis of GERD remains challenging—clinical diagnosis lacks a validated noninvasive biomarker, and BA determination from BALF requires liquid chromatography mass spectroscopy (6). We hypothesized that donor-derived cell-free DNA (dd-cfDNA) from plasma may represent a proxy for detecting BA micro-aspiration and allograft injury. Here, we present a case series of LT patients confirmed negative for rejection and infection and with clinically suspected GERD, who were evaluated to understand the role of dd-cfDNA in monitoring lung function post LT.

## Materials & methods

The transplant program at the Medical College of Wisconsin uses dd-cfDNA for post-transplant surveillance, with tests performed monthly for the first year and every 3 months thereafter. dd-cfDNA fraction (Prospera™ Lung test; Natera, San Carlos) was measured in 5 patients at median 4 weeks pre*-* and post*-* minimally invasive GI surgical (MIGS) intervention for GERD. All blood samples collected for dd-cfDNA testing were drawn in two 10 mL Streck Cell-Free DNA BCT tubes and shipped to the processing laboratory. cfDNA was amplified using massively-multiplexed PCR targeting >13,000 single nucleotide polymorphisms, followed by next-generation sequencing of the resultant amplicons either on the Illumina NextSeq 500 or NovaSeq platforms with a minimum of 8 million reads per sample (7). The dd-cfDNA fraction, analyzed as a percentage of total cfDNA, was reported to the treating physician for use in routine clinical care. Samples with ≥1% dd-cfDNA were considered at increased risk for rejection (8, 9).

**Figure 1:**
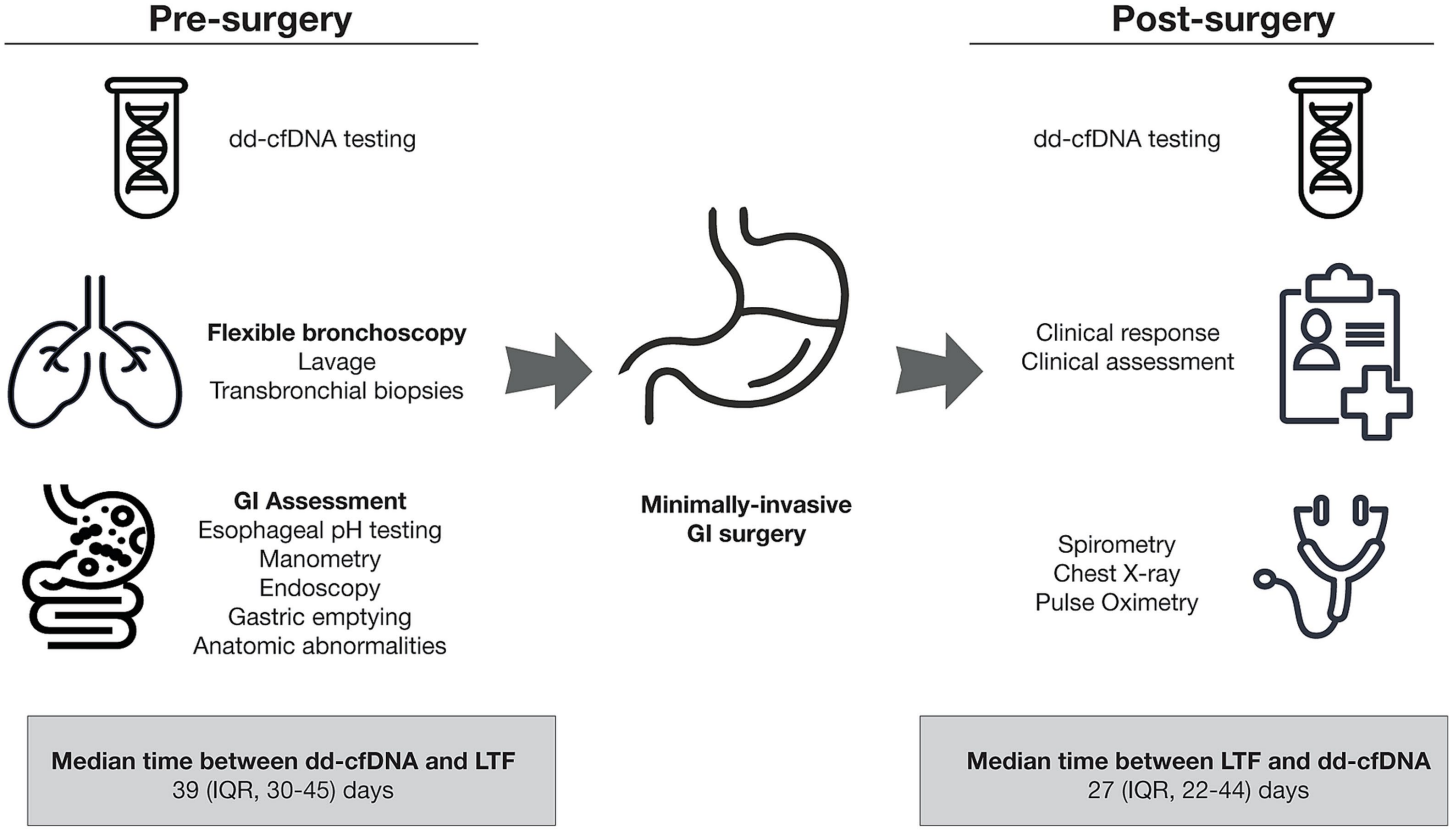
Timeline of testing and surgical procedures.

This study was a quality improvement initiative, with results analyzed retrospectively. LT patients were assessed per site protocol-specified standard-of-care (SoC) with flexible bronchoscopy with trans-bronchial biopsies (TBB) and bronchoalveolar lavage (BAL) at approximately 1-, 3-, 6-, and 12-months, in addition to the clinically indicated procedures for assessment of allograft dysfunction. Patients were evaluated as SoC with an upper gastrointestinal endoscopy, nuclear scintigraphy gastric emptying (Normal: *T*_1/2_ < 90-min), esophageal manometry, esophageal pH impedance investigations to assess indication for laparoscopic Toupet fundoplication (LTF) and potential adjunctive laparoscopic procedures. Clinical outcomes post-MIGS were determined by investigator review of electronic medical records and SoC spirometry results (Figure 1). Patients were only considered for inclusion into this case series if TBB and BAL ruled out both allograft rejection and infection. A multidisciplinary team of minimally invasive surgeons, gastroenterologists, and transplant pulmonologists reviewed patient data monthly. Data are presented as medians (25–75% interquartile range [IQR]) with paired comparisons performed by Wilcoxon matched pair signed rank test (*p* < 0.05).

## Results

Five patients were identified with complete data available pre- and post-MIGS who were transplanted between June 2020 and June 2023 at the Medical College of Wisconsin. Patients received bilateral (*n* = 4) and single LT (*n* = 1; Table 1). Median time post-LT until MIGS procedure was 782 (IQR: 474.5–906) days.

**Table 1:** Patient demographics and characteristics.

Patient ID	Age/sex	Type LT	Indication LT	DMS	MIGS type	Time post-LT (days)	dd-cfDNA pre-MIGS (%)	dd-cfDNApost-MIGS (%)	Clinical outcome
1	57F	BLT	COVID-19 PPF	29.4	LTF, pyloric myotomy	1015	3.27	0.75	↑FVC 400 mL ↑FEV_1_ 400 mL
2	53M	BLT	RA-ILD	23.2	LTF, paraoesophageal hernia repair	259	1.35	0.15	Resolution recurrent aspiration PNA; weaned off O_2_
3	70M	(R) SLT	IPF	NA	LTF, hiatal hernia repair	690	3.24	0.18	Resolved recurrent aspiration PNA; weaned off O_2_
4	69F	BLT	COPD	12.1	LTF	797	14.15	2.9	↑FVC 280 mL ↑FEV_1_ 250 mL
5	53M	BLT	HP	42.5	LTF	782	2	0.75	↑FVC 130 mL ↑FEV_1_ 130 mL
Median (IQR)	57 (53–69.5)			26.3 (17.65–35.95)		782 (474.5–906)	3.24 (1.675–8.71)	0.75 (0.165–1.825)	

BLT, bilateral lung transplant; (R)SLT, right side lung transplant; COVID-19 PPF, SARS-CO(V)-2 progressive pulmonary fibrosis; RA-ILD, rheumatoid arthritis-interstitial lung disease; IPF, interstitial lung disease; COPD, chronic obstructive pulmonary disease/emphysema; HP, hypersensitivity pneumonitis; DMS, DeMeester Score; dd-cfDNA, donor-derived cell-free DNA fraction; LTF, laparoscopic Toupet fundoplication; MIGS, minimally invasive GI surgery; PNA, pneumonia; FVC, forced vital capacity; FEV_1_, forced expiratory volume-1 second.

**Figure 2:**
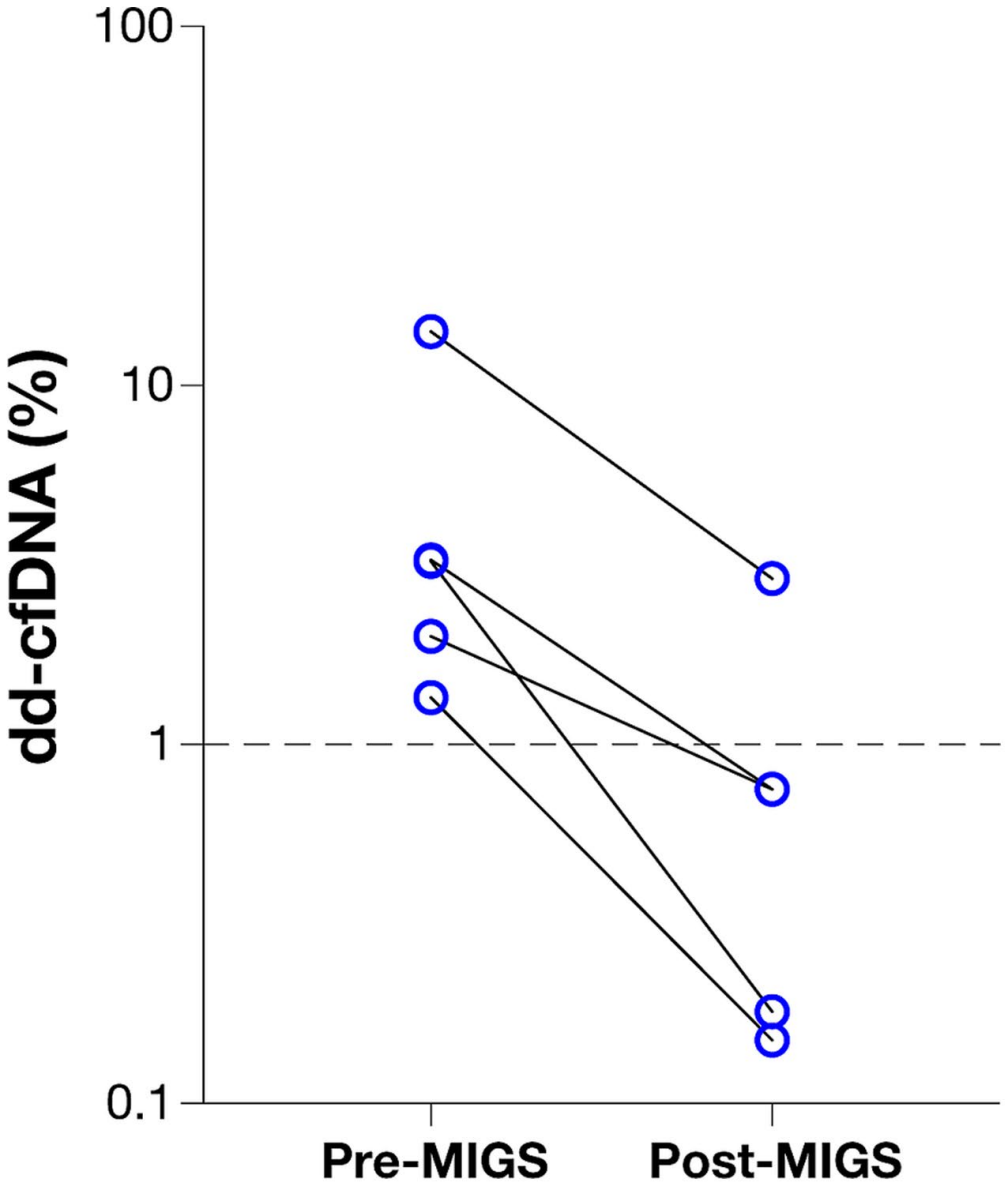
Donor-derived cell-free DNA fraction pre- and post-MIGS for laparoscopic Toupet fundoplication (LTF) and adjunctive surgical procedures. Dotted line represents the threshold for increased-risk of acute rejection (dd-cfDNA% ≥ 1.0%); *y* axis shown in log scale.

Pre-MIGS dd-cfDNA was ≥1% (median 3.24%; IQR, 1.675–8.71%). dd-cfDNA fraction elevation precipitated evaluation by bronchoscopy (inclusive of TBB and BAL procedures), which was conducted within a median of 4-weeks. Histopathologic TBB assessments for all patients were interpreted as Grades A-0 or A-X (no evidence of acute cellular rejection) as per *International Society of Heart and Lung Transplantation* guidelines by an experienced pathologist. BAL microbiology demonstrated enteric pathogens (*E. coli* and *Klebsiella* spp.) in bacterial culture for 1 patient, consistent with a clinical diagnosis of aspiration pneumonitis. Delayed gastric emptying was observed in 3 of 5 patients; all patients had normal esophageal manometry. The DeMeester Score (Normal: <14.7 events) was abnormal (median 26.3; IQR: 17.65–35.95 events) in 3 of 4 patients where this was determined, consistent with a diagnosis of GERD. All five patients underwent a laparoscopic Toupet fundoplication procedure (i.e., partial fundoplication with approximately 270-degree posterior wrap of the stomach around the lower esophagus), with adjunctive procedures including pyloric myotomy in 1 patient and hiatal hernia repair in 2 patients. Clinical improvement was observed in all patients as assessed by spirometry (*n* = 3) or resolution of recurrent aspiration pneumonia hypoxemic events (*n* = 2). dd-cfDNA trended lower post-MIGS in all patients (median 0.75%; IQR: 0.165–1.825%) (*p* = 0.0625), representing a median 77% decrease in dd-cfDNA fraction (Figure 2). The median time between pre-surgery dd-cfDNA assessment and MIGS was 39 (IQR, 30–45) days and between MIGS and post-surgery dd-cfDNA assessment was 27 (IQR, 22–44) days.

## Discussion

The pathobiology of CLAD after LT is highly complex with a myriad of putative immunologic mechanisms (10). An inflammatory and pro-fibrotic milieu in CLAD can lead to alloimmune or autoimmune responses, allograft dysbiosis, and a spectrum of insults, characterized by BA micro-aspiration (5). dd-cfDNA is a clinically validated tool for the detection of acute rejection post-LT, and can serve as an injury biomarker (8, 9). dd-cfDNA elevation has also been observed in the absence of clinical disease, with extreme elevations being identified as a risk factor for subsequent CLAD and mortality (11). The relationship between the frequency of GERD and BA micro-aspiration in cases with cryptic dd-cfDNA elevation is currently unknown.

To our knowledge, this case series reports the first post-LT study of dd-cfDNA levels and clinically determined GERD and BA micro-aspiration in the absence of acute rejection or infection. We posit that the etiology of dd-cfDNA elevation is due to micro-aspiration with pro-inflammatory BA. However, other mechanisms may also be at play, including enzymatic injury due to pepsin, up-regulation of the inflammatory cascade, and alteration in the lung microbiome. A 77% decrease in dd-cfDNA% was seen post-MIGS, suggesting a substantial reduction in molecular injury of the allograft; this was corroborated by clinical improvement after laparoscopic Toupet fundoplication, and improved pulmonary function testing.

While pre-transplant GI evaluation is typically conducted, most patients are not candidates for anti-reflux surgery due to poor health. Reflux testing post-LT is typically delayed until recovery, and evidence of reflux- or aspiration-related graft injury is needed before surgical intervention. Here, elevated dd-cfDNA in the absence of infection or rejection prompted GI evaluation and expedited surgery. Our data suggest that elevation in plasma dd-cfDNA%, when allograft rejection and infection have effectively been excluded, should prompt clinical consideration of foregut dysfunction following LT. Early monitoring and therapy for microaspiration could lead to improved survival among LT patents. It is important to note, however, that the small cohort size along with the heterogeneity of lung-transplant indications, precludes any clinical conclusions, and these results should not be interpreted as definitive evidence of the ability of dd-cfDNA to detect micro-aspiration based allograft injury. Further studies are needed to confirm these findings. Confirmation of these findings in large multi-center clinical trials could prompt an expansion of dd-cfDNA’s utility to include its use as a noninvasive, readily available biomarker for allograft injury due to GERD and BA micro-aspiration. When considering the extremely limited therapeutic options with proven efficacy for CLAD, the availability of a noninvasive clinical tool informing on the molecular sequelae of GERD and BA micro-aspiration offers a unique opportunity for intervention thwarting a prominent determinant in the pathobiology of CLAD.

## Data Availability

The data analyzed in this study is subject to the following licenses/restrictions: This study was a quality improvement initiative, with results analyzed retrospectively. These data are not publicly available. Researchers can reach out to the corresponding author with a request to review deidentified data. Requests to access these datasets should be directed to Samir Sultan (ssultan@mcw.edu).

## Author’s note

A portion of these data were submitted to the 2026 International Society for Heart and Lung Transplantation Congress as an abstract and will be presented as a poster presentation in May 2026.

## Glossary

ALAD: acute lung allograft dysfunction
BLT: bilateral lung transplant
BA: bronchoalveolar
BAL: bronchoalveolar lavage
BALF: bronchoalveolar lavage fluid
CLAD: chronic lung allograft dysfunction
COPD: chronic obstructive pulmonary disease/emphysema
COVID-19 PPF: SARS-CO(V)-2 progressive pulmonary fibrosis
dd-cfDNA: donor-derived cell-free DNA
DMS: DeMeester Score
FEV_1_: forced expiratory volume-1 second
FVC: forced vital capacity
GERD: gastroesophageal reflux disease
HP: hypersensitivity pneumonitis
IPF: interstitial lung disease
IQR: interquartile range
LT: lung transplantation
LTF: laparoscopic Toupet fundoplication
MIGS: minimally invasive GI surgery
PNA: pneumonia
RA-ILD: rheumatoid arthritis-interstitial lung disease
(R)SLT: right side lung transplant
SoC: standard of care
TBB: trans-bronchial biopsies.
